# Health Maxims

**Published:** 1882-06

**Authors:** 


### HEALTH MAXIMS.

The object of the following maxims is to communicate some generally accepted principles in their application to the preservation of health, and the cure of disease without medicine, in short phrase, few words and disconnected sentences; to be taken up and laid down at a moment's notice, on steamship, tramway, packet or rail car, at such odds and ends of time as fall to the lot of travelers and others, and which else might not be appropriated so usefully, because in this age of restlessness and hurry the care of the health, like the search for religion, is considered one of the things which can be dispensed with, until a more convenient season in the future. It is hoped that some who would not spend the time to hear a lecture or read a book may be enticed to peruse a paragraph now and then in reference to the care of the body which, in being put into practice, may have an important bearing in the prolongation of life.

### HEALTH IS A DUTY.

In sickness there is no enjoyment except in the consolations of the Christian religion.

A sound mind in a sound body is a fitting foundation for all that is high and noble in human achievements.

The safest and best remedies in the world, are warmth, rest and abstinence.

Delicious sleep comes oftenest to the young and the day laborer.

A cheerful disposition is the sunshine of the soul.

The mental states have a more controlling influence over the bodily condition than most persons imagine.

There is no better way, no safer way, no easier way, no surer way of saving children from the debasing influences of the street, from corrupting associations, and from the acquisition of vicious and hurtful practices, than to make home attractive.

The education of the young should properly commence with the grandmother, for it takes about two generations to eliminate the plebian from the character and constitution.

Cold is the greatest enemy of old age.

Ventilation is perfect in proportion as the air of an apartment is kept equal in purity to that of the external atmosphere. This is best done in private dwellings by having an open fire-place.

Nature is very much like a shiftless child, who, the more he is helped the more he looks for it. The more medicine a man takes the more he will have to take, whether it be anodyne, tonic or alterative.

The thinnest veil or silk handkerchief thrown over the face while riding or walking against a cold wind is a remarkably comfortable protection.

When alcohol was first introduced into the world in its concentrated form, about the year one thousand, it was called "Aqua Vitæ," the water of life, the great catholicon for human maladies, but it soon became the "Aqua Mortis," the water of death, the source of mortal woes incalculable, hence the curious lines:

"Is 'Aqua' alcohol?
Yes, aquafortis;
'Aqua vitæ' once,
Now 'Aqua Mortis.'"

The most healthful form of exercise is that which involves exhilirating out-door activities.

That old man! what disappointments he has encountered in his long journey, what bright hopes blasted, what sorrows felt, what agonies endured, how many loved ones he has covered up in the grave. And that old woman too! husband dead, children all buried or far away, life's flowers faded, the friends of her youth no more, and she waiting to go soon. Ought we ever miss an opportunity of showing attention to the aged, of proffering a kindness, or lighting up a smile, by a courteous act or a friendly deed?

Many men with a Bible, a Concordance, a Hymn Book, and vigorous health, become more efficient ministers of the gospel than others who, with the advantage of splendid libraries, and the disadvantage of being sickly, have been but cumberers of the ground.

Never sit or stand with the wind blowing on you for a single moment, for it speedily produces a chill, to be followed with a fever and then a bad cold.

If thrown into the water and the strength is failing, turn on the back with only the nose and toes out of the water, hands downward and clasped. This should be practised while learning to swim, as a means of resting from great fatigue in swimming.

We shrink with horror at the thought that we, our wives or our children may possibly die in a mad-house, and yet it can be made impossible by a reasonable attention to the laws of life and health and by an active, stirring life.

To sleep well, a man must work hard.

Exercise to the extent of great fatigue, does more harm than good.

A hearty meal, taken while excessively fatigued, has often destroyed life.

Health and good nature are generally associated.

On a freezing Winter morning, to enter a warm breakfast room, with a blazing fire and a snow white table covering, with cheery faces all around giving hearty welcome, is one of the many domestic felicities of a happy marriage.

The "sands of life" are yielded by the food we eat and the water we drink; they constitute the foundation of the nails and hair and the scales of the skin, for we are all a scaly people, differing from the fish only that our's are smaller, and of variable quantities—morally.

Water is by much the largest constituent of our frames, used to render the other more solid portions plastic; but all decay and die, having been but the casket of the soul, destined for immortality and eternal life.

Chilliness of body dampens the spirits, sours the temper and renders the whole man unlovely.

The comforts and conveniences of life save trouble, save labor, economize time and add to our happiness generally.

A sour look, an impatient gesture, a cross word at the breakfast table is enough to make the best food indigestible and spoils a day.

Cleanliness, in all the surroundings of a family mansion, pays richly in many ways, in good health, moral elevation, personal comfort, and dollars and cents besides.

The ashes of the cremated Lady Dilke weighed just six pounds; so, that after all, our bodies are made up of a few pailfuls of water and a little dust.

A good laugh is anti-dyspeptic.

The wisest men are those who aim to live in such a way as to grow old without aches or pains.

Life is warmth, growth, repair and power to labor, and all these are derived from the food we eat and the fluids we drink, and these should be good.

At every period of life, at all seasons of the year, and from the tropics to the poles, in every clime and country, the temperature of the human body in health is the same to a degree, that is, ninety-eight of Fahrenheit, hence we should eat in Winter mainly of warming food, such as meats, fats, oils, sugar, and all the grains, farinas and starches; in Summer, the fruits and berries, and melons and vegetables of the field, the garden and the orchard, which cool and open, and ventilate the system.

The metals are dissolved by the rains and feed the plants, they in turn feed the animals, and they in turn sustain man, in order to fit him for the duties of time and the rewards of an immortal existence.

Little do the young and vigorous know how the old appreciate those delicate attentions which they so often need in the journey of life, and which it costs so little to bestow, how it cheers their hearts and lifts them up with a delighting thankfulness!

More women than men recover at insane asylums, first, because they have rest; second, in-door life is not so wearing to them, being more accustomed to it.

In a closed sleeping apartment the atmosphere becomes more contaminated every minute, because carbonic acid gas, a deadly poison, is generated in the lungs and is expired at each breath, and combining with the moisture it is heavier than the common air, and settles near the floor, hence, the last thing a man should sell is his bedstead; but in reality it is considered by the ignorant and unfortunate poor as the most dispensible thing in the house, hence sickness is soon added to their poverty, a most unhappy combination.

Divorce, neglect of marriage and the prevention of offspring are crimes against society, against humanity and against the great Creator of us all, and the only efficient method of preventing these increasing and mischievous practices, is in becoming more imbued with the principles inculcated in the Scriptures of the Old and New Testament, to be begun even before the child has learned his A B C, by reading to them the historical portions of the Bible in short lessons and in short explanations, all the while aiming to impress upon the mind and the heart the habit of receiving with implicit confidence, faith and affection, every statement of the Holy Book, as an assertion of the Creator himself, without the most remote thought of calling in question the truth of one single fact or word. Then will none want to go any further than to read His command, "multiply and replenish;" that there is no other sufficient cause for divorce than adultery; and that who ever seeks to baffle the Omnipotent, to circumvent Him in the operation of such physiological laws as He has created, will be found, in the end, to be ruining both body and soul for time and for eternity.

Nine-tenths of the inmates of insane asylums who recover, are those who were sent within a year after the first manifestation of their infirmity.

The best anodyne in all nature, is moderate, steady and continuous exercise in the open air.

It is hard enough to make an honest living in this world, with good health, but to have to work for daily bread in sickness and suffering, is very much like climbing a perpendicular ice bank in frosty weather.

The worst cold may be promptly cured if, within twenty-four hours after it has been taken, the patient will keep warm in bed, and eat little or nothing for a day or two.

More than one-fourth of all the inmates of insane asylums are from the families of farmers and merchants; from the former, because the wives are overworked, and the husbands lack mental culture and variety of occupation, having little to stimulate to mental activities, and a scant knowledge of the laws of health. From the latter in consequence of the reverses of mercantile life. Merchants' families, all over the United States, are among the higher classes, and when they become bankrupt, the mind fails in the attempt to grapple with the difficulties and mortifications of their changed condition, and being without the means to start again in business, and without a trade to compel a support, they soon fall into despondency and discouragement, and the mind topples over.

Never sit with the back to a window or door, even if closed, for the air coming in at crack and crevice, will certainly give a cold.

It is not healthy in any country, at any season of the year, or at any time of life, to get up early, habitually: the old are better rested by lying late, even if not asleep, while the young require all the sleep they can get. In all latitudes, in warm weather, the morning air, although feeling cool and fresh, is laden with the pestiferous miasma In winter the atmosphere, before breakfast, is so cold and chilly and searching, that it fairly shrivels up man and beast, chilling to the very narrow-bone sometimes, hence the average duration of human life would be increased, and the amount of sickness largely diminished, by late, rather than early rising, as all the older nations full well know and practice.

In going out into a colder air, keep the mouth resolutely closed, and walk briskly for a few moments, thus preventing chilliness, which is always the percursor of a cold.

As between husband and wife, that is the nobler spirit which, in difference of opinion, most readily and immediately yields the privilege of the last word to the other party.

As argument in the presence of third persons quickly degenerates into the ignoble ambition of victory, rather than conviction or instruction, and is unprofitable, so is reproof, except when the two are alone; else the admonition is received with impatience, indignation or revenge.

A generous nature never hurts the feelings intentionally.

To remind of a favor is not kind; to speak of it offensively, more than cancels the obligation.

To leave the best for others is generous, to select the best for oneself is the meanest of all traits.

The "gentleman" is magnanimous, the "lady" is serene.

The portion of the body which most requires protection against cold and wind, is that between the shoulder-blades behind, as it is at this point the lungs are attached to the body, and the blood is easily chilled.

To spend two or three moments, on rising and retiring, in rapid frictions of the whole surface of the body with the hand, is a more rational treatment of the skin, and a more health promoting operation, for most persons, than a daily cold water bath.

A good cleansing of the entire body with soap and warm water once a week, is all the bathing the human system requires for purposes of health, in ordinary circumstances.

No rational mind can fail to see that it is a wisdom and a duty to guard against the causes, and watch vigilently against the indications of such diseases as dyspeptia, which often so influences the mind, as to subvert the whole character, making a wreck of happiness, heart and life together.

---

Bitters.—All "Bitters" offered for sale contain Alcohol. Many take them in the place of brandy, whisky, rum and other forms of spirits, persuading themselves that they are reforming as to their beverages. Some bitters have more alcohol in them than whisky Bitters contains as much [illegible] cohol as many kinds of wine and about double the amount contained in various brands of ale. Taking bitters of the mildest kind is the first step towards habitual drunkenness.

### CLARIFYING WATER.

Thirty years ago, in travelling up and down the Missisippi river whose waters, below the mouth of the Missouri, were so turbid that it was impossible to see through a glass, it was a common amusement to tie a bit of alum to a thread and letting it down into the water give it a swinging motion for a minute, and in a few minutes more, the water would be as clear as a rain drop. Within a short time the statement has appeared in the public prints as if it were something new. It may however be well to add the exact proportions in which the alum should be used so as not to make the water taste of it. Three quarters of a pound of finely pulverized alum stirred well into a ton of water ; in smaller quantities to each quart of water, four grains of Alum. The Sulphate of Alumina is greatly better than the Rock or Potash Alum, as it introduces no alkaline matter into the water.

